# Deviations from typical paths: a novel approach to working with GPS data in the behavioral sciences

**DOI:** 10.1186/s12942-022-00305-4

**Published:** 2022-06-18

**Authors:** Karen E. Nielsen, Shannon T. Mejía, Richard Gonzalez

**Affiliations:** 1 Department of Population Health Sciences, School of Public Health, Georgia State University, 140 Decatur St. Suite 400, Atlanta, GA 30303 USA; 2 Department of Kinesiology and Community Health, College of Applied Health Sciences, University of Illinois at Urbana-Champaign, 906 S. Goodwin Ave., Urbana, IL 61801 USA; 3 Department of Psychology and Research Center for Group Dynamics, Institute for Social Research, University of Michigan, Ann Arbor, MI USA

**Keywords:** Intraindividual variability, Deviations, Multi-day studies, Uncertainty in travel behavior, Analytic framework, GPS tracking

## Abstract

**Background:**

Behavioral science researchers are increasingly collecting detailed location data such as second-by-second GPS tracking on participants due to increased ease and affordability. While intraindividual variability has been discussed in the travel literature for decades, traditional methods designed for studying individual differences in central tendencies limit the extent to which novel questions about variability in lived experiences can be answered. Thus, new methods of quantifying behavior that focus on intraindividual variability are needed to address the context in which the behavior occurs and the location tracking data from which behavior is derived.

**Methods:**

We propose deviations from typical paths as a data processing technique to separate individual-level typical travel behavior from a location tracking data set in order to highlight atypical travel behavior as an outcome measure.

**Results:**

A simulated data example shows how the method works to produce deviation measures from a location dataset. Analysis of these deviations offers additional insights compared to traditional measures of maximum daily distance from home.

**Conclusions:**

This process can be integrated into larger research questions to explore predictors of atypical behavior and potential mechanisms of behavior change.

**Supplementary Information:**

The online version contains supplementary material available at 10.1186/s12942-022-00305-4.

## Background

Global Positioning System (GPS) and similar location-tracking technologies allow for collection of rich data concerning travel behavior. Over the last decade there has been growing interest in using the detailed data provided by location tracking to answer research questions in the behavioral sciences dealing with the physical environment individuals inhabit day to day. In particular, activity space and life space are two areas of research that may benefit from such automatic and unobtrusive data collection. For example, concepts that denote the space that an individual occupies in their daily life—such as life space or activity space—were historically measured using self report, but can now be measured using location tracking data [1]. While a variety of metrics have been derived from location data, those currently in use may be limited in representing the full range of life space concepts [2].

Life space and activity space studies, like transportation studies, often summarize data from many consecutive days of measurement into a small number of representative values to approximate tendencies in more granular measures of travel [3–6]. Examples of such measures include the average of each day’s maximum straight-line distance from home or the average area of the space an individual occupies in daily life. Although intraindividual variability in travel behavior is commonly acknowledged [7–9], the focus has primarily been on using multiple days of data to obtain more stable estimates when testing individual differences in central tendencies. In these cases, variation from the individuals’ identified behavior norms is considered error. However, research questions concerning intraindividual variability offer opportunities for new insights into life space and travel behavior. For example, research on individual differences in the number of commute routes used across several days suggests an interest in intraindividual variability in travel behavior among mobility researchers [10]. As multiple days of data are already collected to measure central tendencies, one can use these data to answer new questions about intraindividual variability and atypical behaviors directly.

This intraindividual variability—the day-to-day variability of behavior within persons— allows researchers opportunities to answer new questions about travel behavior and health. These new questions could link day-to-day variation in experiences of health to variation in behavior on that day and allow the magnitude of these associations to vary across person-level characteristics. For example, we would expect life space to not only be smaller among older adults who are more frail [11], but to dynamically shrink on days that older adults report more physical symptoms. Further, as evidence suggests that those with diminished physiological reserve are more sensitive to perturbations in their immediate environments [12], we would expect that this dynamic coupling of life space and physical symptoms would be stronger among frail older adults. In addition to linking experiences on a given day to travel behavior on that day, the magnitude of intraindividual variability in travel behavior may itself be linked to health outcomes. From a dynamic systems perspective, a system will fluctuate as it adapts to change in the person (e.g., changes in health, knowledge, or ability), environment (e.g., change in traffic patterns or road closures), or situation (e.g., new demands such as a new job or a global pandemic) [13]. For example, research on variability in daily time spent on out-of-home activities suggests that non-work activities commonly occur but vary with no single typical amount of time spent across many days [14]. Intraindividual variability in travel behavior could model these activities as deviations from typical work-focused behavior, enabling further exploration into associations with additional behaviors or characteristics of the individual. Further, accounting for the extent of day-to-day variability in transit behavior could provide additional insight in our understanding of passenger segmentation and tailoring of services [15].

A barrier to leveraging the potential of detailed multi-day studies in the framework of intraindividual variation and covariation is that current metrics are not aligned with intraindividual variability-oriented research questions. For example, the daily maximum distance from home, commonly used in lifespace and mobility studies [3, 11, 16–21], could be equal across days that substantially differ in the routes traveled on those days. Such methodological artifacts could mask intraindividual dynamics that link experiences on a given day to travel behavior on that day as well as interindividual differences in the magnitude of day-to-day variability in travel behavior. Previous work has identified periodic behavior of individuals based on frequently visited locations [22, 23] and similarities across individuals based on patterns in routine behaviors [23]. However, these approaches do not address atypical behavior or route variability directly. Existing research on aggregate measures of route-switching behavior assesses the frequency of the various routes used by an individual but does not extract additional features about the intraindividual variation for use in further analyses [10, 24]. Similarly, the common measure of distance from home masks the breadth of behavior, such as side trips and excursions, that occur within a specified distance from home, preventing further study of the variability and motivation for such travel.

In this paper, we outline a general framework for calculating distance from typical paths that can be used to study intraindividual variability in travel behavior based on location tracking data. These measures can be used for further analysis to answer questions about the dynamics of travel behavior and the implications of those dynamics for target outcomes. This framework offers a flexible scaffolding for adaptation to specific research questions concerning deviations from individual-level typical travel behavior. While a variety of measures have been derived from GPS and other location tracking data [2], our approach is novel (to our knowledge) because it focuses on the abnormal measurements that would traditionally be interpreted as noise or errors in the model as a measure of primary interest. Throughout the remainder of the paper, we describe how existing path estimation procedures can be leveraged to provide an individual reference set of routes from which deviations can be computed. These deviations can be quantified in terms of frequency or magnitude for straight-forward inclusion into standard analysis frameworks, such as regression models, to address behavioral questions concerning variability in travel behavior.

We begin with a step-by-step guide for implementing this framework, including the two-stage procedure of estimating an individual’s typical path and computing deviations from that path. The framework allows for substantial modification to tailor the approach to specific research questions. An example with simulated data follows, demonstrating how this framework can be applied and modified. Using this example, we compare our approach to a common distance from home technique to illustrate the utility of our deviation from typical path technique to capture day-to-day variability in travel behavior. We additionally provide a demonstration of the application of the proposed path estimation technique to real-world data. We end with a discussion of the framework’s utility and possible directions for future applications and developments in this area.

## Methods

We outline a framework involving six steps, presented in Table 1. These steps apply to both primary and secondary data analysis. The first three steps, which typically occur prior to data collection, involve formalizing questions, definitions, and assumptions and include: (1) establishing a research question, (2) establishing theory and formalizing assumptions, and (3) operationalizing the target dynamic and determining a priori groupings of data. The second three steps represent our primary contribution and outline the process of analyzing location tracking data after it has been collected: (4) defining and determining typical paths, (5) calculating deviations, and finally (6) analyzing deviations. Each step is described sequentially in detail below.

**Table 1:** Steps for deviations from typical paths framework

Formalizing questions, definitions, and assumptions		
Step 1	Establish a research question	The research question facilitates the process of formalizing the definition of a typical travel behavior and the purpose for studying variation in travel behavior.
Step 2	Establish theory and formalize assumptions of the target dynamics of travel behavior	Theory is used to characterize the behavioral dynamics under question including the timescale over which the travel behavior unfolds and behavior cycles.
Step 3	Operationalize target dynamic and determine a priori groupings of data	Develop the timeframe of interest (e.g., morning commute or combined daily commutes) and grouping (e.g., weekday commute and weekend trips) units based on theorized habitual travel behavior and potential day to day variability in that behavior.
Analysis of location tracking data		
Step 4	Defining and determining typical paths	Estimate or define a typical path for each grouping within person—e.g., if the target dynamic is theorized to differ on weekdays and weekends, two separate typical paths should be obtained for each person.
Step 5	Calculate deviations	One deviation is calculated per observation using a distance metric to find the distance from the observed point to the typical path.
Step 6	Analyze deviations	Analysis should reflect the research question, theory, and groupings established. Multilevel models are well-suited to intraindividual studies of variation, with time-varying covariates allowing for study of covariation over theorized timescales.

### Formalizing questions, definitions, and assumptions

#### Establish a research question

Before estimating a typical path and computing deviations, we must first establish a research question that defines both the typical travel behavior we are trying to capture and the purpose for studying deviations from this typical behavior. Researchers must decide, for example, whether the study aims to examine individual differences in the magnitude of variability, the representativeness of a typical path, or the association of transit behavior on a given day to experiences on that day. Researchers must also consider whether the focus is on temporal variability, spatial variability, or both. The chosen research question must align with the data used. For example, questions focused on intraindividual variability in daily travel behavior will require multiple days of data to address. The research question will also be critical in selecting an appropriate path estimation procedure, quantification of deviations, and analysis.

#### Establish theory and formalize assumptions of the target dynamics of travel behavior

The second step is to establish the appropriate theory to characterize the behavioral dynamics under consideration. Theory is essential at this step in the process because it orients the research to the temporal dynamics of the travel behaviors, which are critical to decisions involving data collection and processing. For example, before data processing begins, researchers must decide on the timescale over which behaviors unfold (seconds, minutes, hours, days, weeks) and their cycle for repetition (hours, days, weeks). Researchers may realize that the behavior unfolds across nested times scales, such as momentary behaviors nested within a day, nested within day of the week, nested within the season. Additionally, researchers must refer to or develop a theory to dictate whether a meaningful growth process (e.g., shrinking or expansion of life space or travel behavior during the study period) is also expected. For example, increased travel away from an established location or route could be reflective of a growing flexibility or freedom to travel, or it may indicate an increased need to obtain resources that the currently-accessed built environment does not readily provide [25]. Theory can also establish whether the dynamics of the target travel behavior allow for a typical path to exist. Some researchers have suggested, for example, that there is not a typical day of non-commute travel [14]—but perhaps modeling the commute travel as the typical behavior could provide meaningful context to the non-commute travel that allows for new insights. In cases when data are already collected, exploratory analyses or corresponding qualitative reports may provide inspiration for establishing inherent dynamics.

Due to the flexibility of our technique, it is essential to consider the dynamics of the travel behavior prior to estimating typical paths and deviations. This will assist in defining which controls and constraints are necessary to discover informative deviations in travel behavior. For example, does a lunch excursion from work count as a deviation that is worthy of study? Is a change in common travel behavior, such as a stop at a gas station, a meaningful deviation? Is a route change, such as traffic detour, of interest? When possible, establishing these analysis goals prior to data collection allows researchers to ask participants for these key times or locations to facilitate easier data processing. Either way, clear definitions for target dynamics of travel behavior can improve precision of the estimated path, which will improve estimates of deviations.

#### Operationalize target dynamic and determine a priori groupings of data

Now that the theoretical framework has been established, data processing decisions can be made to organize the data to align with the target dynamic. Similar to establishing theory, it is preferable to make these decisions prior to data collection when possible. However, adjustments can be made after data collection to accommodate unanticipated behaviors in the data. First, researchers need to decide how to best group the observations based on anticipated travel behavior. A typical path will be estimated for each group of observations, meaning that the groups should reflect the shared travel behavior. A typical path should have an established scope, based on start and end location, time of day, or both. The groupings therefore define identifiable and repeatable behaviors across the observed time series.

We note that the duration of a path may differ across studies. For example, a full-day path is considered relevant for life space, but a study on commute patterns may focus on distinct commutes to and from work. Within a study, multiple typical paths such as weekday and weekend paths may exist for each person. Depending on the study, these categorizations may be established prior to data processing as context- or hypothesis-driven, or they may be explored during processing and analysis as data-driven groupings. In this paper, we will focus on a priori established groupings. For example, studies concerning commute-oriented travel should identify days on which the individual commutes. Given the increased availability of work-from-home options, a longer period of data collection may be needed to identify the typical path for individuals who commute less often. Similarly, individuals who travel to multiple workplaces will require their commutes to be grouped by workplace.

Second, after groupings of observations have been established, the data need to be organized to reflect the theory and align with the path estimation procedure in statistical software. Long format, in which each observed location is a separate row, will be needed in most cases. If separate groupings, such as weekdays and weekends, exist and correspond to separate typical paths to be estimated, the observations for each group should be maintained in separate datasets or a single dataset with clear labels that can be used for subsetting prior to path estimation and again to assign points to the appropriate typical path when calculating deviations.

Once these steps concerning research goals and data processing have been completed, the procedure for obtaining typical paths and deviations from data can commence. The remaining analytical steps, including technical details and modifications, are described in the sections below. As these steps represent the primary contribution of this paper, each step is assigned its own section.

### Defining and determining typical paths

The fourth step in this framework is defining and determining typical paths. For our purposes, a path is a continuous mapping of the unit interval onto the two-dimensional space defined by latitude and longitude coordinates, otherwise known as a plane curve. Existence of a typical path is a critical assumption for our framework, and prior steps leading to the organization of observations into groups must establish groupings for which a typical path is believed to exist. Each grouping of observations will correspond to an estimated path. This definition can be extended to the three-dimensional space including time if deviations in timing are of primary or additional interest. Alternatively, timestamps can be used to define groupings and covariates in analysis, rather than used as a third dimension during path estimation. Specific features of the path can be encoded in the definition and estimation procedure. For example, closed paths with the same starting and ending points allow for treating the entire day as the grouping because we can assume that the individual starts and ends each day or trip at a common location. As mentioned above, other groupings of observed location points are possible, such as focusing on the commute to work. In this case, we would instead fit a curve that is not closed to the data with known starting point at home and end point at work. Assumptions such as limiting travel to known roads are possible to incorporate via alternative path estimation techniques such as map-matching, lending face validity to the estimated path. It is also possible to replace the estimated continuous path in this framework with a dense set of discrete ordered or unordered points defined as latitude, longitude pairs representing all geographic locations that the individual passes through as part of their route.

The process of determining a typical path can be done in any number of different ways, and depends on the design of the study and data collection. Below, we describe principal curves as a data-driven option, followed by a brief note on the use of self-reported typical paths. We also offer potential adjustments to account for study design and research goals. Alternative approaches to estimating a typical path can be substituted in this modular framework.

#### Data-driven typical paths—principal curves and variations

Estimating a path is essentially fitting a curve with no gaps or discontinuities through the data. This process should be completed separately for each grouping if multiple typical paths need to be estimated—for example, typical paths for weekdays and weekends should be estimated separately from separate datasets. There are several options for fitting curves to location data to produce an estimated travel path, most of which are considered smoothing procedures in the statistics literature. We recommend selecting a smoothing procedure based on matching known properties of the data with the assumptions of the procedure. For example, when travel is known to be limited to roads, additional steps could be taken to limit the estimated typical path to known roads. Within this paper, we focus primarily on the use of principal curves to estimate a typical path for two reasons. First, they are relatively simple and easy for behavioral researchers without prior experience in the analysis of GPS data to use via implementations in common statistical software. Second, they facilitate estimation of a typical path over the entire course of study by simply including all data points for all relevant days or trips when estimating a curve.

Principal curves are a nonlinear generalization of principal components, or equivalently a variation on nonlinear regression that allows for symmetric treatment of variables when minimizing errors [26]. Conceptually, each location on the curve is the average of all nearby data points—in this case, all nearby observed location coordinates. While principal curves utilize a similar least squares criterion as linear regression, they minimize squared deviations in all variables compared to only the response variable in regression frameworks. This fits well with the unsupervised learning goal of path estimation [27].

In the context of travel behavior, a principal curve is a nonparametric smoother that reflects a typical path traveled by an individual. Multiple technical definitions of principal curves and corresponding algorithms have been offered by various authors [26, 28–33]. We make use of the approach described by [26] and implemented in the princurve package in R [34]. This approach begins with the principal component line and follows a standard Expectation-Maximization (EM) approach, iterating between projection of an updated curve and calculation of conditional expectation until the change in total squared distances from all points to the curve is less than a prespecified threshold. We make use of smoothing splines fit using generalized cross-validation during the expectation step, though a variety of options for smoothers exist and can be substituted. Further adjustments to improve the estimated path after fitting a principal curve are described at the end of this section.

An important limitation of applying the principal curves framework to travel behavior is that the resulting simple curves cannot intersect themselves. As a result, some travel patterns that naturally occur over the course of a trip or day may be impossible to estimate. This type of travel behavior may be more likely in dense urban environments. There are several possibilities for addressing this limitation. One option is to use finer-grain segmentation of observations to develop subgroups for fitting portions of the typical path at a time, resulting in a collection of principal curves defined based on shorter trips or trip segments that can be nested within group for analysis. Another option is to make use of an alternative smoother. For example, strategies to locally fit principal curves could resolve these situations [32, 33]. The princurve package in R [34] offers a choice of periodic LOWESS for fitting closed curves that could reflect travel in a loop. A third option is to incorporate time or temporal ordering of observed points as a third variable to prevent the self-intersecting that occurs in two dimensions due to crossing back over a segment later in time—such as returning on the same route as departing, or taking a cloverleaf highway interchange. Following the estimation of a path over time, the path can be reduced to two dimensions for computations of deviations if temporal deviations are not of interest, or the path can remain in three dimensions to allow for the study of temporal deviations in addition to location deviations.

In addition to principal curves, several options have been proposed in the literature for estimating and smoothing a path from point-based location data, such as splines and functional data analysis techniques [35], hidden Markov models [36], and Kalman filters [37]. All of these statistical smoothing techniques require selection of one or more tuning parameters that describe the interval of time over which to smooth and order of the assumed underlying functional form (in the case of spline- and kernel-based smoothers) or the amount of noise anticipated in the measurement and overall process (in the case of Kalman filters) [37]. Principal curves similarly require decisions concerning tuning parameters; however, many modifications to the principal curve algorithm have been proposed that allow for flexibility in adapting the framework to a variety of situations. For this reason, we have chosen to focus on principal curves as a technique that is broadly applicable—though other techniques may be preferable given specific assumptions concerning travel behaviors. Regardless of the choice of smoothing technique, the estimated typical path should be graphed and assessed visually for goodness of fit to the observed points and to ensure that the estimated typical path is sensible in light of established theory of travel behavior.

Once a typical path has been estimated, researchers may choose to use additional techniques to make adjustments to the estimated typical path that improve precision and add context. Such optional tasks can complement the smoothing procedure to provide the most accurate typical path possible, though they should be done carefully to avoid overfitting. One such technique is map matching, which makes use of known features of the geographic space, such as roads and traffic patterns, to refine estimated travel patterns [38]. Map matching techniques treat locations as nodes connected by directed edges in graphs which can be matched to known nodes and road segments in an established map. Several researchers have proposed map matching algorithms to define or improve estimated paths [39], particularly in low-sampling-rate settings [40]. Although these techniques can increase the accuracy of the estimated path for individual trips under the assumption that all travel is along known roads, they require a reference library of road networks. Alternative principal curve fitting techniques can also be used to improve the fit by bringing in additional data—for example, [41] offer an adaptive algorithm for principal curve estimation that incorporates known endpoints, allowing for specification of known start and end points to trip groupings that can improve fit of the path near the end points.

#### Self-reported typical paths

We note that self-reports of typical travel routes provide an alternative to data-driven path estimation. Self-reported typical paths can also be treated as a complement to data-driven paths for validating paths or comparing findings—for example, in studies interested in recall. These routes can be stored concisely as turn-by-turn directions or an ordered list of waypoints (e.g., intersections of roads) prior to analysis. Depending on the prior established grouping, it may be necessary to collect multiple routes per person. When obtaining self-reported typical paths, it can be helpful to have local maps available and ask follow-up questions to validate their reflections on typical travel behaviors. Questions such as “Do you have any stops that you typically make along the way?” or “Do you take different routes if there is traffic?” can validate recall, and questions such as “Do you also make this trip on weekends?” can assure that no participant-specific groupings have been missed. Once typical paths have been established, either using data-driven techniques or self-report, researchers can proceed to the next step of computing deviations.

### Computing deviations

Once the typical travel routes have been defined, the next step is to calculate distances from the associated typical path for every observed location point. As with estimation of a typical path, the step of recording deviations is modular and may be replaced or built upon with alternatives to suit the specific application. Here, we highlight use of Euclidean distance to quantify distance of each observation to the estimated path.

We begin with a collection of points belonging to a particular estimated path. The most straightforward organization is to subset the observed points based on the same grouping for which typical paths have been estimated—for example, when separate paths are estimated for weekdays and weekends, calculate deviations for weekday points based on the weekday path and deviations for weekend points based on the weekend path. The points in a given grouping should not belong to multiple paths (i.e., each point can belong to a weekday or a weekend but not both), but can belong to only a portion of a longer typical path depending on interpretation goals. For example, deviations for only a morning commute can be calculated and studied using a path based on travel for an entire day.

Each point is assigned a quantification of its deviation from its associated typical path via a distance measure. In practice, the nearest location to the path can be found by densely sampling along the estimated path and using the nearest path point to the observed point for calculating distance. Often the estimated path will already be stored as a dense sampling of points in statistical software, and this process can be automated by calculating the distance from the observed point to all path points and taking the minimum. Distance can reflect a spatial deviation such as traveling to a new location, a temporal deviation such as a traveling to an established location at a new time, or both—depending on whether time was included in the estimation of the typical path and in which dimensions the distance is calculated.

The simplest version of a deviation is a spatial deviation based on Euclidean distance, to produce a distance “as the crow flies” from the observed point to the typical path. Further adjustments, such as using a Haversine distance or map projection to adjust for the curvature of the earth, are possible [42]—though in many cases these adjustments are trivial in comparison to the measurement error in real-world passive location tracking. Distance may also be measured using travel distance along roads or estimated travel time to obtain a more practical measure of the movement that would be required to resume travel along the typical path from the current point.

### Analysis of deviations

The final step of this framework is analysis of deviations to gain insights into atypical behavior. Once distances have been computed for every point, they can be analyzed, alone or with complementary information, using a variety of methods. If data were separated into multiple datasets for path estimation and calculation of deviations, they may need to be recombined into a single dataset with retained labels prior to analysis.

The process of computing deviations results in a calculated deviation for every observed point. Depending on the sampling rate of the GPS device and distance traveled, this could mean hundreds or thousands of values for every travel behavior grouping (e.g., day) defined for analysis. These rich data allow for a variety of analytic approaches. Deviations for individual sampled points can be used directly in multilevel models, growth curve models, or other techniques focused on intraindividual variance and covariance based on the research question [43]. These models address the longitudinal, repeated measures design of location-tracking studies, as well as the clustering of observations within day and within person—making them a natural fit for many research questions concerning variability in travel. Additional steps could be taken to identify atypical destinations by including timestamps and clustering observations that deviate from the estimated path.

Summary measures can also be derived to indicate individual differences in travel behaviors for use as either independent or dependent variables. For example, the maximum deviation, total or average deviation, or number of deviation clusters in a grouping may also be useful quantities to estimate. The average of all deviations can be interpreted as a measure of “goodness of fit” for the estimated typical path(s), where a larger average deviation suggests additional behavior that is not captured by the typical path(s), error in measurement such as noisy GPS data, or both. Daily average deviations can describe how typical each day was. In our example below, we demonstrate the utility of such summary measures, as well as the importance of selecting a summary measure that aligns with project goals. The analytic approach should be chosen to match the research protocol and reflect the established theory in terms of grouping, timescale, and frequency of behavior cycle. The approach should also be robust to any data quality concerns such as missing data and irregular sampling rates that occur with some collection methods.

## Results

We now illustrate our approach using a simulated example investigating a person’s travel flexibility. Our aims in presenting this example are first to illustrate the deviations from typical paths technique and second to compare this technique to a traditional distance-from-home approach to measuring daily travel behaviors. By using simulated data, we are able to highlight performance of the path estimation and summary of deviations in a known context. For the purpose of our example we simulated data to represent an individual’s travel behavior observed from passive GPS location tracking on a cell phone over a 50-day time period. Our simulated data reflect a usual commute route as well as trips to several other destinations, but for analysis we did not know the person’s work schedule, travel modality, or typical route to work and other common destinations. We assumed that the person went to the same workplace regularly and based their daily travel from a specific home location. We expected to identify a typical path that largely reflected the commuting route, allowing us to interpret any perturbations from that route as reflections of flexibility in travel—one of the possible interpretations offered by [25] for observed increases in mobility. We evaluated our method by comparing it to other standard approaches to mobility. We end with a demonstration of the proposed path estimation technique on real-world data to show the capabilities and limitations of the principal curves procedure in this context.

### Description of simulated data

We simulated location data for a single individual over the course of 50 days using the Google Directions API [44] accessed via the googleway R package [45]. This person traveled from home each day. On most days, the person traveled from home to work and back along the same route. On other days, the person traveled from home to work to a specific store and back home, or from home to a non-work destination and back. The key locations were selected in a midwestern city by the researchers to make use of realistic geography and travel patterns forced by roads. The route for each travel pattern suggested by the API was used without digression. Observation timestamps were not used in this simulation.

After routes were generated by the Google Directions API, they were converted to latitude and longitude coordinates using the R package googleway [45] and routes were repeated to reflect 50 days with patterns occurring in the following proportions: 70% of days followed a usual commute from home to work and back without additional stops (“commute days”); 20% of days were usual commutes that involved an additional stop at a store while returning to home from work (“commute-and-shop days”), and 10% of days involved travel to one of 5 other destinations outside the home (“destination days”; 2%, or one day, each). Within each day, points were sampled along the selected path, on average 350 m apart, reflecting the irregularity with which a location tracking device such as a smartphone would collect location data. For commute days, this resulted in an average of 213 observations per day. To further simulate the noise in typical smartphone location data, small independent perturbations were added to both the latitude and longitude coordinates. These perturbations were normally distributed with mean 0 and standard deviation of .002 degrees. In the selected geographic area, .002 degrees is approximately 220 ﻿meters north or south, and 160 meters east or west. Example R code for simulating data is available in Additional file 1﻿.

### Application of method to simulated data

With our simulated data, we walk through the steps of applying our method to the simulated data. Our research question was “How much does a daily commute to work deviate from its typical route?”

The second step is to formalize theory concerning the research question and available data. We determined that each observed location coordinate served as an observed measure of travel behavior and that these observations were nested within a commute to and from work. We further assumed that work and home locations were stable over the observed period of time. The commute was a behavior that unfolded over the entire day to include both the morning and evening commute, and this behavior repeated daily on most days with no additional nesting of the behavior cycle such as weekday and weekend behavior. We assumed stability in the behavior over the course of the study period, with no growth or constriction in the commute or deviations. A typical path was expected to exist and reflect the favored commuting route of the individual.

We next began the third step of operationalizing the target dynamics and establishing the groupings that determine our data processing decisions. Given that neither our research question nor theory concerned specific segments of a day, and instead treated commute behavior as a process that unfolds over the course of the entire day, we retained all GPS observations. Each day served as a grouping over which the typical path was estimated. There were no known or theorized higher-level groupings of days, such as weekdays and weekends, so a single path was estimated as the reference travel behavior for all days. For data organization, we retained samples in long format wherein each observed location was a row, with columns for latitude, longitude, study day, and simulated route type. Typically, route type labels would be unknown—however, grouping labels such as weekday and weekend may exist based on a priori hypotheses. To demonstrate our approach, we summarized deviations using these route labels.

**Fig. 1:**
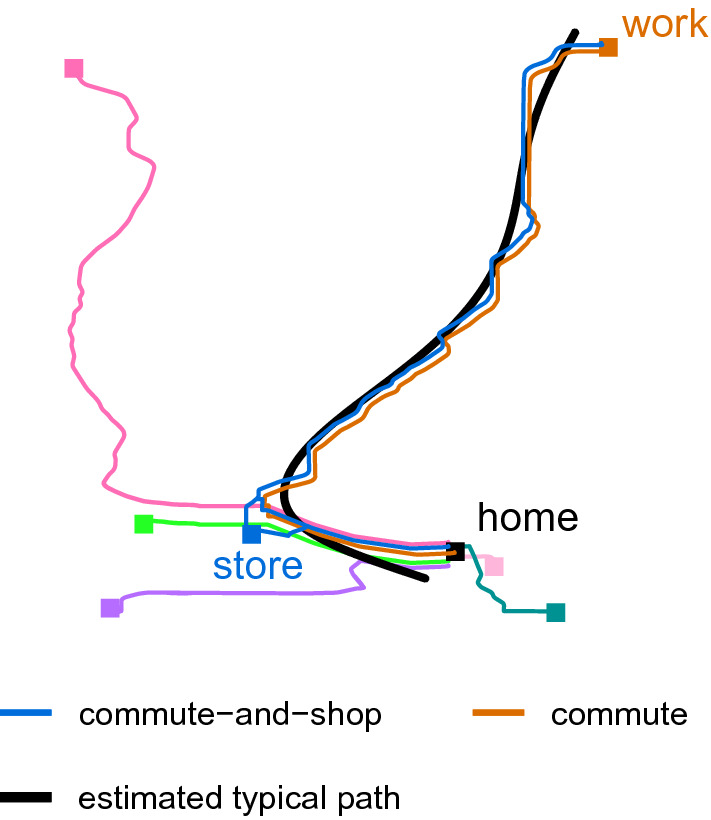
All simulated routes and estimated typical path. Jitter used to show overlapping lines. There are 5 destinations visited once each, along with the more common commute route (70% of days) and commute-and-shop route (20% of days). The locations of home, work, and the store visited during the commute-and-shop route are labeled.

We then applied the two-stage procedure of extracting meaningful information from our data beginning with the fourth step, estimating a single principal curve using all points from all 50 simulated study days to uncover a single data-driven typical path. For this example we estimated the principal curve using the princurv package in R [34]. Figure 1 shows the 7 routes from which data were sampled, along with the estimated typical path. From this figure, we can see that the estimated typical path closely followed the usual commuting route, smoothing over the small bends. No additional processing was done after fitting the principal curve, so this estimated typical path was not constrained to follow roads. To provide a comparison of deviations obtained from an ideal map-matched typical path, we included the true commute route as an alternative to the estimated typical path.

**Fig. 2:**
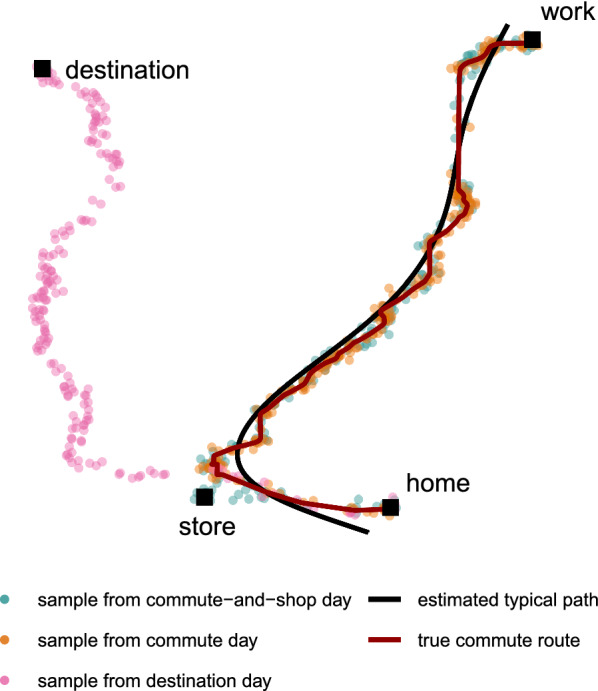
Select observed locations for three types of days. Estimated typical path is superimposed on the true usual commute route and points from three different days—a commute day, a commute-and-shop day, and a destination day. All stops are labeled.

Figure 2 shows this typical path superimposed on points from three different days, with each point’s color reflecting the type of travel pattern from which it was sampled. It is easy to visually distinguish the route taken on the destination day, but the commute day and commute-and-shop day are less distinguishable.

We next obtained deviations from the path for every observed point and stored these values as an additional variable associated with each observed point in the data set. Due to the principal curve fitting procedure, each observed point used in the estimation of a typical path had a corresponding projection onto the final curve. Using the observed location, as well as the projection, we computed a distance for each observation. We similarly computed minimum distance from each observed point to the known commute route in addition to the estimated typical path. For this demonstration we computed Haversine distances, which are similar to Euclidean distances but account for the curvature of the earth, using the distHaversine function within the R package geosphere [42]. We chose this approach for computing deviations to show an incremental build on simple Euclidean distance. Example R code for estimating a principal curve and computing deviations is available in Additional file 2﻿. Alternative curve fitting procedures may require alternative steps, such as densely interpolating the typical path curve to identify projections corresponding to each observation.

**Fig. 3:**
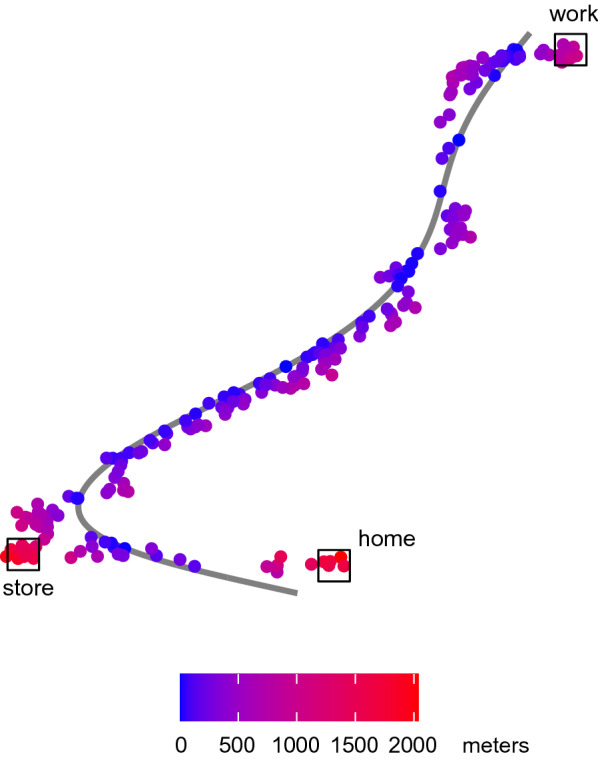
Commute-and-shop day observations shaded by deviation from typical path. Observed locations recorded from a single commute-and-shop day are shaded based on their calculated deviations in meters from the typical path estimated using principal curves, which is shown as a solid line. All stops are labeled. The largest deviations are observed around the store stop and home endpoint.

At this point, we note that by calculating the deviations from the estimated typical path for the commute-and-shop day from Figure 2, we can identify the excursion to the store systematically to assist with analysis and interpretation. Figure 3 shows the points from the selected commute-and-shop day, shaded based on their calculated deviations from the estimated typical path. We can see that deviations were high around the store, highlighting potential flexibility in travel behavior on this day that allowed for this excursion. There were also large deviations around home. This was true for every day due to the gap between the end of the estimated typical path and the home location—demonstrating that any misfit in the typical path estimation can have downstream impacts. Visualizations such as this one are critical for both interpretation and quality checks for model misfit. We can also see that there was noise in the observed points, resulting in some points along the estimated path having larger deviations while others have smaller deviations. Additional data processing could be undertaken to reduce this measurement noise, but could also have unintended consequences such as removing or attenuating true deviations.

The final step in our framework is analysis of the deviations. When analyzing travel behavior across many individuals, we suggest using multilevel models, growth curve models, or other approaches that focus on intra-individual variability. For this simple example consisting of data for only one individual, we limited our analysis to creation of summary measures of the average and maximum deviation per day. Table 2 shows the mean and standard deviation of these summary measures for each type of day and compares deviations obtained from the estimated typical path and the true commute route. We can see that both summary measures reveal differences in the extent of deviation from the associated path for the three types of day, with the smallest calculated deviations belonging to the commute days and largest deviations belonging to the destination days. The variability in deviations was similar between the commute and commute-and-shop days. Findings for the estimated typical path aligned with our expectation that the estimated typical path would reflect the usual commute route, and provide insights into our research question (“How much does a daily commute to work deviate from its typical route?”). It appears that there was minimal deviation in the daily commute on days when the individual only commuted, increased deviation on days when the individual included a stop to shop during their commute, and greatest deviation on days when the travel was not true commute behavior. The deviation values were smaller when using the true commute route as a reference, demonstrating the imperfect estimation of the typical path using principal curves. However, consistent patterns in the summaries of deviations were visible across the two reference path options.

**Table 2:** Summary measures of average and maximum deviation per type of day

		Commute (n = 35)		Commute-and-shop (n = 10)		Destination (n = 5)	
		Mean	sd	Mean	sd	Mean	sd
Estimated typical path	Average	425	13	499	14	3460	2197
	Maximum	1805	148	2044	149	6190	3767
True commute route	Average	161	6	226	6	2855	2425
	Maximum	566	87	1601	127	5492	3971

Summarizing deviations (meters) from both the estimated typical path and true commute route within each day uncovered anticipated trends in travel behavior for this individual. Specifically, the greatest deviations were found on the destination days and smallest deviations were found on commute days. Shopping trips were reflected with increased deviations on commute-and-shop days relative to commute-only days. Smaller deviations were recorded when using the true commute route as the reference path. sd = standard deviation of the daily measured deviations.

### Comparison to existing methods

**Fig. 4:**
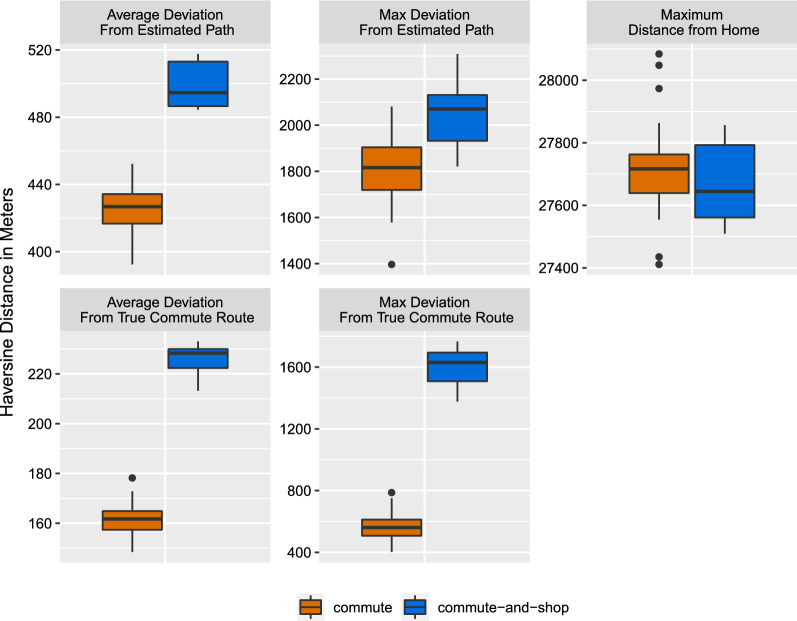
Differential separation of deviations on commute days with and without shopping across competing summary measures. For both the estimated typical path and the true commute route, using the average of all calculated deviations from a typical path provides the greatest separation between two similar types of days—a day in which the person only commutes and a day in which they commute and shop—while the traditional measure using a single distance referenced to a single point offers minimal separation. Separation is greater when using the true commute route as a reference rather than the estimated typical path. In this comparison, Haversine distances in meters are used for all measures but the range of values displayed differs across the graphs. The emphasis is on the differing separation between day types within each graph, rather than comparison of each type of day across graphs.

An additional aim of this paper is to show that our deviation from typical path technique is superior to the standard distance from home measure in answering research questions about variability in travel behavior. Figure 4 compares deviations to maximum distance from home, a common life space measure. We summarized deviations from both the estimated typical path and the true commute route separately to further compare these two options. In order to establish a common timescale to allow for comparison of these measures, we first summarized deviations within each day to obtain a single value of either maximum deviation (the single largest deviation value observed that day) or average of all deviations per day, along with computing the maximum distance from home using the original observations.

This simple simulation shows that studying deviations from a typical path estimated from data can recover information about anomalous travel behavior under favorable conditions, such as normally distributed measurement errors in observed points, adequate sampling density, and a clear prevailing travel behavior that meets the characteristics of a principal curve for estimating the typical path.

### Application of path estimation using real-world data

**Fig. 5:**
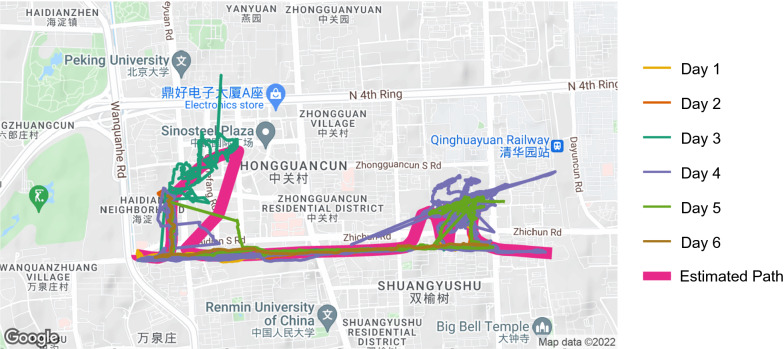
Principal curve typical path estimation applied to real-world data. Estimated typical path and recorded travel behavior from six days for a single person.

To demonstrate that our proposed data-driven typical paths approach (step 4) is possible using real-world data, we selected 6 days with similar travel behavior for a single individual from the GeoLife GPS Trajectories data set [46–48], estimated a typical path using principal curves with LOWESS smoother, and computed daily maximum and average distance from the estimated typical path. Code for this demonstration is available in Additional file 3﻿. Figure 5 shows that it is possible to estimate a typical path that approximates the prevailing observed travel behavior with minimal impacts from measurement error. The example also displays a limitation of using a single principal curve to estimate travel in certain real-world scenarios: this individual appears to backtrack on their travel near the western edge of the map, and a single principal curve cannot contain such a loop. However, the average deviations for each day summarized in Table 3 are relatively small, indicating that the estimated path does not deviate substantially from the observed points. Table 3 also highlights the day-to-day variability captured by the average and maximum deviations calculated for each day—for example, deviations are higher on average on day 3 compared to the other five days.

**Table 3:** Summary measures of average and maximum deviation per day

Day	Average deviation	Maximum deviation	Observations
1	20.20	125.07	1281
2	24.91	124.31	1141
3	45.29	591.90	3311
4	27.98	619.43	23285
5	21.51	214.26	6201
6	21.74	124.57	880

Summarized deviations from the estimated typical path show that a single principal curve can closely approximate observed travel behavior over several days while highlighting day-to-day variability.

## Discussion

GPS and other location-tracking technologies have facilitated convenient and affordable collection of travel behavior of individuals, which in turn enables research into behavioral patterns that can address important questions concerning topics spanning public health, urban design, traffic routing, and others. While existing work has established the utility of location tracking for such questions [1] and offered a variety of metrics for analysis of such data [2], we note that relatively little work has developed methods focused on intraindividual variability despite a demonstrated need [7–10]. Instead, existing approaches tend to summarize behavior into highly aggregated measures of travel and routine behavior [3–6, 22, 23]. We offer a framework that models not only the prevailing behaviors of individuals, but also emphasizes atypical travel behavior that can provide novel insights into intraindividual variability and change. The deviations estimated within our framework can be used to summarize individual differences in the dynamics of travel behavior or as time-varying phenomena that are linked to daily experiences. Within our modular framework, we provided guidelines for both data-driven and self-reported definitions of typical paths, and outlined the process for calculating deviations from the established path. We showed that our method captures day-to-day variability in travel behavior that is masked by the common distance from home measure.

### Results discussion

Through our results section, we provided an example of the application and utility of the proposed framework. Using a simulated dataset that contains 50 days of primarily commuting behavior, we showed how a typical path that reflects the prevailing commute behavior can be estimated using a principal curve. We showed how deviations measured from this estimated path compare to those measured from the true commute route. The use of simulated data provided the benefit of known labels for each day and known commute behavior to demonstrate performance of the method using suggested path estimation and deviation computation options. We additionally demonstrated that the principal curve approach can be applied to real-world travel data and highlighted the additional complexities and data quality issues that may require case-by-case modifications of the approach to address.

Our procedure captures meaningful travel behavior because travel away from the typical path is visible as distinct clusters of high-deviation points without additional processing. For example, larger deviations on days with travel away from the typical path indicate meaningful travel behavior beyond the measurement noise and inaccuracy in the estimated path captured on commute-only days. This demonstrates that meaningful information can be extracted even with a simple path estimation procedure such as principal curves on unprocessed data. However, our visualization of the calculated deviations relative to the estimated typical path revealed that even days that are commute-only, and should thus match the typical path, have non-zero deviation. This can be attributed to two sources of variability—measurement noise of the points and inaccuracy in the estimated path. We demonstrated that deviations are lower when using the known commute route as a reference, which eliminated the inaccuracy of the estimated path. Thus, pre-processing steps such as map-matching [38–40] can improve both typical path estimation and observed locations used for calculating deviations.

We additionally showed how the deviations can be studied to provide new insights. Our comparison of simple summary measures shows not only that averaging by type of day recovers anticipated trends with larger deviations coming from commute-and-shop days, but that the deviations can be used to identify atypical behavior that occurs nearby established behavior. For example, the stop at the store is missed when measuring travel behavior using the maximum distance from home. This highlights a key innovation of our proposed framework: updating the reference from a single established point, such as home, to using established travel behavior as measured by the typical path as the reference. Our findings suggest that current approaches may underestimate flexibility in travel behavior, and our proposed framework offers potential for new insights by additionally capturing the variety of travel that may occur between the reference location and the most distant locations.

Our example of path estimation using principal curves applied to real-world data mirrors findings based on simulated data. The calculated deviations were small overall, suggesting that this estimated typical path may be adequate for research questions concerning atypical travel behavior such as visits to new destinations or new route choices. Additional situation-specific steps, such as segmenting the travel behavior or including temporal ordering, could be taken to more closely model the individual-level typical travel behavior prior to calculating deviations.

While our examples used summary measures to show the differential separation achieved by orienting each observation to a path rather than a single point, the calculated deviations could also be used directly in other modeling frameworks such as multilevel models. The advantages of using the deviations directly in analyses include allowing every observation to provide unique information and having many measurements per day rather than a single measure, allowing us to consider variability across many timescales. Critically, we demonstrated that deviations from a typical path provide different information about travel behavior than common existing measures—more specifically, they describe how unusual travel behavior is, rather than how distant travel is from a set location. This opens new questions about atypical travel and travel flexibility that were previously limited by available methodologies.

### Method discussion

We proposed a novel framework for the analysis of location tracking data which highlights deviation in travel behavior as a meaningful behavioral indicator. This approach allows for direct analysis of day-to-day variability within individuals and identification of atypical travel behavior. Existing work on the analysis of location tracking data has focused primarily on summarizing typical travel behavior and treats deviations as noise [2]. These existing approaches, such as the distance from home and lifespace ellipse, mask the variety of travel behavior that occurs between the reference location and most distant locations. Our proposed emphasis on deviations from typical paths informs understanding of inter- and intra-individual variability throughout travel behavior and can be achieved using passive location tracking possible with existing technology such as smartphones.

This framework provides substantial flexibility in the estimation of both paths and deviations, making it adaptable to a variety of research questions. In particular, multiple approaches can be taken to both estimate typical paths and compute deviations from those paths. Typical paths can be estimated with a variety of algorithms such as the principal curves method described here, or with procedures that incorporate additional information such as limiting to roads via map matching. Alternative smoothers or approaches to data-driven path estimation may work well in specific situations and for specific research questions. The principal curves approach documented here offers a flexible, data-driven option that fits a curve for each person, without the need for specialized tools or software. Our method allows for data analysis even in cases where knowledge of the geography and built environment is unknown.

Importantly, the principle curves approach we describe does not preclude self-reported or otherwise pre-defined typical paths. Instead, such predefined paths can be used either as an alternative or complement to data-driven typical paths when estimating deviations. We advocate for the data-driven approach because it minimizes participant burden and recall bias. Additionally, the data-driven approach easily accommodates secondary analysis of existing tracking data where additional questions cannot be asked of the individuals represented in previously-collected data.

Our technique is also flexible in that typical paths can represent specific trips unique to an individual, daily routes of an individual, or common behavior of grouped individuals. Similarly, deviations can be estimated using Euclidean distance, or additional information such as known streets can be used to estimate a true travel distance for vehicular transportation. Further, this framework can be applied across many scales—while the examples in this paper focus largely on travel via vehicles on streets, the approach can be applied to understand movement within a single building such as an office space or long term care facility.

### Limitations and future directions

Our framework provides substantial flexibility for identifying atypical travel behavior and flexibility in travel behavior. This enables researchers to gain new insights into intraindividual variability. However, the potential of this method must be viewed within the context of its limitations. First, we acknowledge that our formal model assumptions may limit application in specific circumstances. For example, the framework would be challenging to apply if a typical path did not exist. Indeed, prior research has found a lack of typical travel behavior within non-commute travel [14], highlighting the need to ensure that the research question and context support a typical path assumption. Further, realistic behavior often includes paths with loops, which violates assumptions for spatial fitting of many path estimation techniques including the principal curves highlighted here. Potential solutions for violations of these assumptions could include modifications to theorized target dynamics and groupings for data organization to ensure that the theory and research question align with using this technique.

We also note that real-world passive location tracking data, such as smartphone location data, also introduces challenges that may impact applications of this framework. On the technology side, gaps in the data and irregularities in sampling rate are common with smartphone location data [49], particularly in dense urban environments. The context of this missing data will determine how impactful the data loss is. For example, regularly missing observations along a straight stretch of highway where deviations are either unlikely or impossible will have minimal impact on the estimation of the typical path and the results. However, missing observations in a dense urban area where there are many opportunities for side trips can lead to failure to identify true deviations. Our method does provide some robustness in the path estimation for occasional missing data due to the longitudinal collection of observations on behavior that repeats many times over the course of a study. Addressing missing data will likely require situationally-specific solutions, but general approaches that weight some observations more than others or interpolate location may improve both the typical path estimation and analysis of deviations. Alternative approaches to estimating the typical path, such as map matching, may provide improvements in these contexts. Future work should explore applications to real-world data, including study of reliability of typical path estimation, and detail situation-specific solutions.

Finally, an important area for future work would be to address the patterns that occur in deviation measures and violate assumptions of downstream analysis approaches. Unlike deviations along the typical path, which should be small and reflect noise in measurement and the estimated path, consecutive deviation values away from the typical path will exhibit autocorrelation. Incorporating use of time stamps is one possible approach to identifying these patterns. Time series techniques may be useful for addressing autocorrelation while retaining all values. Alternatively, measures traditionally used for unprocessed location tracking data [2] may be applied here by changing the reference from a single point to the typical path. For example, the time spent away from the path may be a relevant summary measure of the deviation. The further study of these and other summary measures based on deviations from typical paths, including temporal deviations, is an area for future adaptation and study of their statistical properties.

## Conclusions

In conclusion, our framework emphasizes deviations in travel behavior to enable novel research concerning flexibility and anomalies in travel behavior across a variety of content areas. The framework facilitates answering new questions about travel behavior and health by leveraging the increased ease and affordability of densely sampled passive location tracking of individuals. Researchers have substantial flexibility to fine-tune the approach to specific research questions and specific contexts, and we offer initial suggestions that are broadly applicable throughout our step-by-step guide to facilitate ease of implementation.

## Supplementary Information

**Additional file 1.** Simulate Data.

**Additional file 2.** Process Analyze Data.

**Additional file 3.** Real Data Example.

## Data Availability

Example code for generating and analyzing data is included in the Additional files.

## Abbreviations

API: Application Programming Interface
EM: Expectation-maximization
GPS: Global positioning system
LOWESS: Locally weighted scatterplot smoother

## References

[1] Fillekes MP, Röcke C, Katana M, Weibel R (2019). Self-reported versus gps-derived indicators of daily mobility in a sample of healthy older adults. Soc Sci Med.

[2] Fillekes MP, Giannouli E, Kim EK, Zijlstra W, Weibel R. Towards a comprehensive set of GPS-based indicators reflecting the multidimensional nature of daily mobility for applications in health and aging research. Int J Health Geograph. 2019;18(17). 10.1186/s12942-019-0181-0. 10.1186/s12942-019-0181-0 PMC6657041 31340812

[3] Boissy P, Blamoutier M, Brière S, Duval C (2018). Quantification of free-living community mobility in healthy older adults using wearable sensors. Front Public Health.

[4] Hirsch JA, Winters M, Clarke P, McKay H. Generating GPS activity spaces that shed light upon the mobility habits of older adults: a descriptive analysis. Int J Health Geograph. 2014;13(51). 10.1186/1476-072X-13-51. 10.1186/1476-072X-13-51 PMC4326206 25495710

[5] Schenk AK, Witbrodt BC, Hoarty CA, Carlson RH, Goulding EH, Potter JF, Bonasera SJ (2011). Cellular telephones measure activity and lifespace in community-dwelling adults: proof of principle. J Am Geriatr Soc.

[6] Wan N, Qu W, Whittington J, Witbrodt BC, Henderson MP, Goulding EH, Katrin Schenk A, Bonasera SJ, Lin G (2013). Assessing smart phones for generating life-space indicators. Environ Plann B Plann Des.

[7] Pendyala RM, Pas EI. Multi-day and multi-period data for travel demand analysis and modeling. TRB Transportation Research Circular E-C008: Transportation Surveys: Raising the Standard. 2000;1–28.

[8] Pas EI (1986). Multiday samples, parameter estimation precision, and data collection costs for least squares regression trip-generation models. Environ Plann A.

[9] Pas EI, Koppelman FS (1987). An examination of the determinants of day-to-day variability in individuals’ urban travel behavior. Transportation.

[10] Li H, Guensler R, Ogle J (2005). Analysis of morning commute route choice patterns using global positioning system-based vehicle activity data. Transport Res Record J Transport Res Board.

[11] Baker PS, Bodner EV, Allman RM (2003). Measuring life-space mobility in community-dwelling older adults. J Am Geriatr Soc.

[12] Clegg A, Young J, Iliffe S, Rikkert MO, Rockwood K (2013). Frailty in elderly people. Lancet.

[13] Diehl M, Hooker K, Sliwinski MJ (2015). Handbook of intraindividual variability across the life span.

[14] Kang H, Scott DM (2010). Exploring day-to-day variability in time use for household members. Transport Res Part A Policy Pract.

[15] Egu O, Bonnel P (2020). Investigating day-to-day variability of transit usage on a multimonth scale with smart card data. A case study in Lyon. Travel Behav Soc.

[16] Long J, Reuschke D. Daily mobility patterns of small business owners and homeworkers in post-industrial cities. Comput Environ Urban Syst. 2021;85(101564). 10.1016/j.compenvurbsys.2020.101564.

[17] Sun S, Folarin AA, Ranjan Y, Rashid Z, Conde P, Stewart C, Cummins N, Matcham F, Dalla Costa G, Simblett S, Leocani L, Lamers F, Sørensen PS, Buron M, Zabalza A, Guerrero Pérez AI, Penninx BW, Siddi S, Haro JM, Myin-Germeys I, Rintala A, Wykes T, Narayan VA, Comi G, Hotopf M, Dobson RJB, RADAR-CNS Consortium (2020). Using smartphones and wearable devices to monitor behavioral changes during COVID-19. J Med Internet Res.

[18] Giannouli E, Bock O, Mellone S, Zijlstra W. Mobility in old age: capacity is not performance. BioMed Res Int. 2016;(2016). 10.1155/2016/3261567. 10.1155/2016/3261567 PMC4789440 27034932

[19] Stalvey BT, Owsley C, Sloane ME, Ball K (1999). The life space questionnaire: a measure of the extent of mobility of older adults. J Appl Gerontol.

[20] Bowling CB, Muntner P, Sawyer P, Sanders PW, Kutner N, Kennedy R, Allman RM (2014). Community mobility among older adults with reduced kidney function: a study of life-space. Am J Kidney Dis.

[21] Peel C, Sawyer Baker P, Roth DL, Brown CJ, Brodner EV, Allman RM (2005). Assessing mobility in older adults: the UAB study of aging life-space assessment. Phys Ther.

[22] Li Z, Ding B, Han J, Kays R, Nye P. Mining periodic behaviors for moving objects. In: Proceedings of the ACM SIGKDD International Conference on Knowledge Discovery and Data Mining. 2010;1099–1108. 10.1145/1835804.1835942.

[23] Lv M, Chen L, Chen G (2013). Mining user similarity based on routine activities. Inf Sci.

[24] Hatcher SG, Mahmassani HS (1992). Daily variability of route and trip scheduling decisions for the evening commute. Transp Res Rec.

[25] Susilo Y, Kitamura R (2007). Analysis of day-to-day variability in an individual’s action space: exploration of 6-week mobidrive travel diary data. Transport Res Record J Transport Res Board.

[26] Hastie T, Stuetzle W (1989). Principal curves. J Am Stat Assoc.

[27] Brunsdon C. Path estimation from GPS tracks. In: Proceedings of the 9th International Conference on GeoComputation. 2007.

[28] Tibshirani R (1992). Principal curves revisited. Stat Comput.

[29] Kégl B (2000). Learning and design of principal curves. IEEE Trans Pattern Anal Mach Intell.

[30] Verbeek JJ, Vlassis N, Kröse B (2002). A k-segments algorithm for principal curves. Pattern Recogn Lett.

[31] Hastie T, Tibshirani R, Friedman JH (2009). The elements of statistical learning.

[32] Delicado P (2001). Another look at principal curves and surfaces. J Multivar Anal.

[33] Einbeck J, Tutz G, Evers L (2005). Local principal curves. Stat Comput.

[34] Cannoodt R. princurve 2.0: Fit a Principal Curve in Arbitrary Dimension. CRAN. 2018. 10.5281/zenodo.3351282. https://github.com/rcannood/princurve.

[35] Hu X, Yuan Y, Zhu X, Yang H, Xie K (2019). Behavioral responses to pre-planned road capacity reduction based on smartphone GPS trajectory data: a functional data analysis approach. J Intell Transport Syst Technol Plann Opera.

[36] Ozdemir E, Topcu AE, Ozdemir MK (2018). A hybrid HMM model for travel path inference with sparse GPS samples. Transportation.

[37] Jun G, Guensler R, Ogle JH (2006). Smoothing methods to minimize impact of global positioning system random error on travel distance, speed, and acceleration profile estimates. Transport Res Record J Transport Res Board.

[38] Yildirimoglu M (2021). Joint estimation of paths and travel times from Bluetooth observations. Transportmetrica B.

[39] Quddus MA, Ochieng WY, Noland RB (2007). Current map-matching algorithms for transport applications: state-of-the art and future research directions. Transport Res Part C Emerg Technol.

[40] Lou Y, Zhang C, Zheng Y, Xie X, Wang W, Huang Y. Map-matching for low-sampling-rate GPS trajectories. GIS. In: Proceedings of the ACM International Symposium on Advances in Geographic Information Systems, 2009;352–361. 10.1145/1653771.1653820.

[41] Zhang J, Chen D, Kruger U (2008). Adaptive constraint K-segment principal curves for intelligent transportation systems. IEEE Trans Intell Transp Syst.

[42] Hijmans RJ, Williams E, Vennes C. Spherical trigonometry: Package ’geosphere’. 2019. https://CRAN.R-project.org/package=geosphere.

[43] Hershberger SL, Moskowitz DS (2013). Modeling intraindividual variability with repeated measures data: methods and applications.

[44] Google Directions API. https://developers.google.com/maps/documentation/directions/overview.

[45] Cooley D. googleway: Accesses Google Maps APIs to Retrieve Data and Plot Maps; 2020. https://CRAN.R-project.org/package=googleway.

[46] Zheng Y, Xie X, Ma W-Y. Mining interesting locations and travel sequences from gps trajectories. In: Proceedings of International Conference on World Wide Web 2009 (2009). https://www.microsoft.com/en-us/research/publication/mining-interesting-locations-and-travel-sequences-from-gps-trajectories/.

[47] Zheng Y, Li Q, Chen Y, Xie X, Ma W-Y. Understanding mobility based on gps data. In: Proceedings of the 10th International Conference on Ubiquitous Computing. UbiComp ’08, pp. 312–321. Association for Computing Machinery, New York, NY, USA; 2008. 10.1145/1409635.1409677.

[48] Zheng Y, Xie X, Ma W-Y. Geolife: a collaborative social networking service among user, location and trajectory. IEEE Data Eng Bull. 2010.

[49] Shen L, Stopher PR (2014). Review of GPS travel survey and GPS data-processing methods. Transport Rev.

